# 2-Chloro-4-{(*E*)-[(4-chloro­phen­yl)imino]­meth­yl}phenol

**DOI:** 10.1107/S1600536812005193

**Published:** 2012-02-10

**Authors:** Zarife Sibel Şahin, Şamil Işık

**Affiliations:** a Department of Physics, Faculty of Arts and Sciences, Ondokuz Mayıs University, Kurupelit, TR-55139 Samsun, Turkey

## Abstract

In the title Schiff base compound, C_13_H_9_Cl_2_NO, the dihedral angle between the mean planes of the benzene rings is 10.20 (10)°. The crystal structure is stabilized by O—H⋯N hydrogen bonds and weak π–π stacking inter­actions [centroid–centroid distance = 3.757 (1) Å].

## Related literature

For Schiff bases related to coordination chemistry, see: Calligaris *et al.* (1972 ▶); Cozzi (2004 ▶); Curini *et al.* (2002 ▶). For the anti­bacterial, anti­cancer, anti­inflammatory and antitoxic properties, see: Williams (1972 ▶); Karia & Parsania (1999 ▶); Desai *et al.* (2001 ▶). For the industrial and biological properties of Schiff bases, see: Lozier *et al.* (1975 ▶); Aydogan *et al.* (2001 ▶). For structural studies of Schiff bases, see: Gül *et al.* (2007 ▶); Şahin *et al.* (2005 ▶); Şahin, Ağar *et al.* (2009 ▶); Şahin, Erşahin *et al.* (2009 ▶); Şahin, Işık *et al.* (2009 ▶). For the classification of hydrogen-bonding patterns, see: Bernstein *et al.* (1995 ▶).
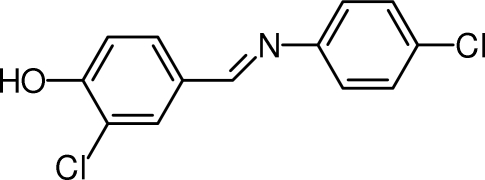


## Experimental

### 

#### Crystal data

C_13_H_9_Cl_2_NO
*M* *_r_* = 266.11
Orthorhombic, 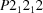

*a* = 9.7438 (6) Å
*b* = 9.9953 (5) Å
*c* = 12.1342 (6) Å
*V* = 1181.78 (11) Å^3^
*Z* = 4
Mo *K*α radiation
μ = 0.53 mm^−1^
*T* = 296 K
0.42 × 0.34 × 0.24 mm

#### Data collection

Stoe IPDS II diffractometer
Absorption correction: integration (*X-RED32*; Stoe & Cie, 2002 ▶) *T*_min_ = 0.807, *T*_max_ = 0.901
11473 measured reflections
2325 independent reflections
1960 reflections with *I* > 2σ(*I*)
*R*_int_ = 0.049

#### Refinement

*R*[*F* ^2^ > 2σ(*F* ^2^)] = 0.031
*wR*(*F* ^2^) = 0.064
*S* = 0.96
2325 reflections
154 parameters
H-atom parameters constrained
Δρ_max_ = 0.12 e Å^−3^
Δρ_min_ = −0.17 e Å^−3^
Absolute structure: Flack (1983 ▶), 968 Friedel pairs
Flack parameter: 0.02 (6)

### 

Data collection: *X-AREA* (Stoe & Cie, 2002 ▶); cell refinement: *X-AREA*; data reduction: *X-RED32* (Stoe & Cie, 2002 ▶); program(s) used to solve structure: *SHELXS97* (Sheldrick, 2008 ▶); program(s) used to refine structure: *SHELXL97* (Sheldrick, 2008 ▶); molecular graphics: *ORTEP-3 for Windows* (Farrugia, 1997 ▶); software used to prepare material for publication: *WinGX* (Farrugia, 1999 ▶).

## Supplementary Material

Crystal structure: contains datablock(s) I, global. DOI: 10.1107/S1600536812005193/jj2120sup1.cif

Structure factors: contains datablock(s) I. DOI: 10.1107/S1600536812005193/jj2120Isup2.hkl

Supplementary material file. DOI: 10.1107/S1600536812005193/jj2120Isup3.cml

Additional supplementary materials:  crystallographic information; 3D view; checkCIF report

## Figures and Tables

**Table 1:** Hydrogen-bond geometry (Å, °)

*D*—H⋯*A*	*D*—H	H⋯*A*	*D*⋯*A*	*D*—H⋯*A*
O1—H1⋯N1^i^	0.82	1.96	2.778 (2)	176

Symmetry code: (i) 
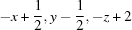
.

**Fig. 1.**
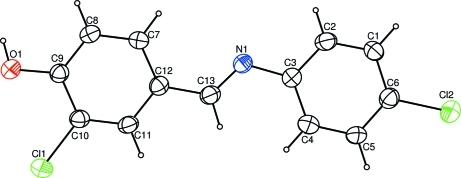
The molecular structure of the title compound, showing the atom-numbering scheme. Displacement ellipsoids are drawn at the 30% probability.

**Fig. 2.**
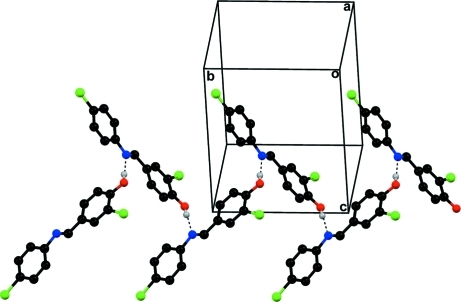
Molecular packing of the title compound, viewed along the b axis. O—H···N hydrogen bonds are shown as dashed lines. H atoms not involved in these interactions have been omitted for clarity.

## supplementary crystallographic information

### Comment

Schiff bases are important in diverse fields of chemistry and biochemistry owing to their biological activites (Calligaris *et al.*, 1972; Lozier *et al.*, 1975). Most Schiff bases have antibacterial, anticancer, antinflammatory and antioxic properties (Williams, 1972). The present work is part of our structral study of Schiff bases (Gül *et al.*, 2007; Şahin, Ağar *et al.*, 2009; Şahin, Işık *et al.*, 2009; Şahin, Erşahin *et al.*, 2009) and we report here the structure of the title compound, C_13_H_9_Cl_2_NO, (I).

The dihedral angle between the mean planes of the two aromatic rings is 10.20° and the C12—C13—N1—C3 torsion angle is 175.96 (17)° (Fig. 1). All bond lenghs are within normal values. The N1—C13 double bond length (1.268 (2)Å) is similar to the corresponding bond lengths in *E*-2-Methoxy-6-[(2-trifluoromethylphenylimino) methyl]phenol (1.270 (5) Å) (Şahin *et al.*, 2005) and *E*-4-Methyl-2-[3-(trifluoromethyl)-phenyliminomethyl]phenol (1.270 (3) Å] (Gül *et al.*, 2007).

In the crystal, the molecules are linked into sheets by O—H···N hydrogen bonds (Table 1) generating C(8) chains (Bernstein *et al.*,1995) along (011)(Fig. 2). Weak, symmetry independent π–π stacking interactions are observed which may influence crystal stability . The perpendicular distance from *Cg*1 to *Cg*1^ii^ [symmetry code: (ii) = -*x*, 1 - *y*, *z*] is 3.62 (0)Å. The centroid-to-centroid distance is 3.757 (1)Å.

### Experimental

The title compound, (I), was prepared by refluxing a solution mixture containing 3-chloro-4-hydroxybenzaldehyde (0.008 g 0.051 mmol) in 20 ml ethanol and 4-chloroaniline (0.007 g 0.051 mmol) in 20 ml ethanol for 1 h. Crystals of (I) suitable for X-ray analysis were obtained from ethyl alcohol by slow evaporation (yield %54; m.p 441–442 K).

### Refinement

All H atoms were placed in calculated positions and constrained to ride on their parents atoms, with CH = 0.93Å and OH (hydroxyl) = 0.82Å with *U*_iso_(H)=1.2*U*_eq_(C, O).

### Crystal data

**Table:**

C_13_H_9_Cl_2_NO	*F*(000) = 544
*M**_r_* = 266.11	*D*_x_ = 1.496 Mg m^−^^3^
Orthorhombic, *P*2_1_2_1_2	Mo *K*α radiation, λ = 0.71073 Å
Hall symbol: P 2 2ab	Cell parameters from 20026 reflections
*a* = 9.7438 (6) Å	θ = 1.7–27.3°
*b* = 9.9953 (5) Å	µ = 0.53 mm^−^^1^
*c* = 12.1342 (6) Å	*T* = 296 K
*V* = 1181.78 (11) Å^3^	Prism, yellow
*Z* = 4	0.42 × 0.34 × 0.24 mm

### Data collection

**Table:**

Stoe IPDS II diffractometer	2325 independent reflections
Radiation source: fine-focus sealed tube	1960 reflections with *I* > 2σ(*I*)
Graphite monochromator	*R*_int_ = 0.049
Detector resolution: 6.67 pixels mm^-1^	θ_max_ = 26.0°, θ_min_ = 1.7°
ω scans	*h* = −11→12
Absorption correction: integration (*X-RED32*; Stoe & Cie, 2002)	*k* = −12→12
*T*_min_ = 0.807, *T*_max_ = 0.901	*l* = −14→14
11473 measured reflections	

### Refinement

**Table:**

Refinement on *F*^2^	Secondary atom site location: difference Fourier map
Least-squares matrix: full	Hydrogen site location: inferred from neighbouring sites
*R*[*F*^2^ > 2σ(*F*^2^)] = 0.031	H-atom parameters constrained
*wR*(*F*^2^) = 0.064	*w* = 1/[σ^2^(*F*_o_^2^) + (0.0363*P*)^2^] where *P* = (*F*_o_^2^ + 2*F*_c_^2^)/3
*S* = 0.96	(Δ/σ)_max_ = 0.003
2325 reflections	Δρ_max_ = 0.12 e Å^−^^3^
154 parameters	Δρ_min_ = −0.17 e Å^−^^3^
0 restraints	Absolute structure: Flack (1983), 968 Friedel pairs
Primary atom site location: structure-invariant direct methods	Flack parameter: 0.02 (6)

### Special details

**Table:**

Geometry. All e.s.d.'s (except the e.s.d. in the dihedral angle between two l.s. planes) are estimated using the full covariance matrix. The cell e.s.d.'s are taken into account individually in the estimation of e.s.d.'s in distances, angles and torsion angles; correlations between e.s.d.'s in cell parameters are only used when they are defined by crystal symmetry. An approximate (isotropic) treatment of cell e.s.d.'s is used for estimating e.s.d.'s involving l.s. planes.
Refinement. Refinement of *F*^2^ against ALL reflections. The weighted *R*-factor *wR* and goodness of fit *S* are based on *F*^2^, conventional *R*-factors *R* are based on *F*, with *F* set to zero for negative *F*^2^. The threshold expression of *F*^2^ > σ(*F*^2^) is used only for calculating *R*-factors(gt) *etc*. and is not relevant to the choice of reflections for refinement. *R*-factors based on *F*^2^ are statistically about twice as large as those based on *F*, and *R*- factors based on ALL data will be even larger.

### Fractional atomic coordinates and isotropic or equivalent isotropic displacement parameters (Å^2^)

**Table:**

	*x*	*y*	*z*	*U*_iso_*/*U*_eq_	
Cl1	−0.02767 (6)	0.22741 (7)	0.73432 (4)	0.06816 (19)	
Cl2	0.78712 (7)	1.02829 (7)	0.54617 (5)	0.0792 (2)	
O1	0.01370 (14)	0.20491 (15)	0.96945 (11)	0.0596 (4)	
H1	0.0307	0.1983	1.0354	0.089*	
N1	0.42961 (15)	0.66841 (17)	0.80818 (13)	0.0474 (4)	
C12	0.25372 (18)	0.4980 (2)	0.82414 (15)	0.0491 (5)	
C9	0.09145 (18)	0.3021 (2)	0.92525 (15)	0.0475 (5)	
C11	0.16101 (18)	0.4218 (2)	0.76339 (16)	0.0532 (5)	
H11	0.1528	0.4365	0.6880	0.064*	
C6	0.6818 (2)	0.9216 (2)	0.62138 (17)	0.0562 (5)	
C8	0.1838 (2)	0.3779 (2)	0.98593 (16)	0.0568 (5)	
H8	0.1924	0.3631	1.0613	0.068*	
C13	0.33844 (19)	0.5943 (2)	0.76529 (17)	0.0541 (5)	
H13	0.3245	0.6022	0.6897	0.065*	
C1	0.68403 (19)	0.9260 (2)	0.73602 (17)	0.0562 (5)	
H1A	0.7417	0.9850	0.7730	0.067*	
C10	0.08170 (19)	0.3259 (2)	0.81251 (15)	0.0487 (5)	
C2	0.5999 (2)	0.8419 (2)	0.79293 (17)	0.0596 (6)	
H2	0.6014	0.8442	0.8695	0.072*	
C4	0.5127 (2)	0.7514 (2)	0.62570 (17)	0.0675 (6)	
H4	0.4552	0.6925	0.5885	0.081*	
C5	0.5955 (2)	0.8342 (2)	0.56650 (18)	0.0685 (6)	
H5	0.5940	0.8320	0.4899	0.082*	
C7	0.2623 (2)	0.4739 (2)	0.93676 (16)	0.0527 (5)	
H7	0.3227	0.5241	0.9794	0.063*	
C3	0.51177 (17)	0.75259 (19)	0.74034 (15)	0.0469 (4)	

### Atomic displacement parameters (Å^2^)

**Table:**

	*U*^11^	*U*^22^	*U*^33^	*U*^12^	*U*^13^	*U*^23^
Cl1	0.0696 (3)	0.0808 (4)	0.0541 (3)	−0.0187 (3)	−0.0139 (2)	−0.0002 (3)
Cl2	0.0909 (4)	0.0874 (4)	0.0592 (3)	−0.0312 (3)	0.0115 (3)	0.0010 (3)
O1	0.0660 (8)	0.0659 (9)	0.0468 (7)	−0.0123 (7)	0.0010 (6)	0.0061 (7)
N1	0.0488 (9)	0.0486 (10)	0.0448 (9)	0.0033 (8)	0.0035 (7)	0.0006 (8)
C12	0.0462 (10)	0.0534 (12)	0.0477 (11)	0.0029 (9)	0.0022 (8)	0.0021 (9)
C9	0.0450 (9)	0.0526 (12)	0.0447 (10)	−0.0002 (9)	0.0026 (8)	0.0009 (9)
C11	0.0550 (10)	0.0667 (13)	0.0379 (10)	−0.0020 (10)	−0.0040 (9)	0.0050 (10)
C6	0.0606 (12)	0.0551 (13)	0.0528 (12)	−0.0015 (11)	0.0068 (9)	−0.0004 (10)
C8	0.0636 (12)	0.0680 (14)	0.0387 (11)	−0.0075 (11)	−0.0017 (9)	0.0044 (9)
C13	0.0536 (11)	0.0650 (13)	0.0438 (11)	0.0004 (10)	−0.0004 (9)	0.0027 (10)
C1	0.0545 (11)	0.0591 (13)	0.0549 (12)	−0.0096 (10)	−0.0027 (10)	−0.0073 (10)
C10	0.0464 (10)	0.0563 (13)	0.0433 (10)	0.0008 (9)	−0.0038 (8)	−0.0006 (9)
C2	0.0635 (12)	0.0741 (16)	0.0413 (11)	−0.0017 (12)	−0.0010 (9)	−0.0027 (10)
C4	0.0827 (14)	0.0689 (16)	0.0510 (12)	−0.0199 (13)	−0.0014 (11)	−0.0100 (12)
C5	0.0920 (15)	0.0728 (16)	0.0406 (11)	−0.0281 (13)	0.0017 (11)	−0.0082 (11)
C7	0.0552 (10)	0.0596 (13)	0.0432 (10)	−0.0070 (10)	−0.0054 (9)	−0.0026 (10)
C3	0.0482 (9)	0.0446 (11)	0.0480 (10)	0.0051 (8)	0.0021 (8)	−0.0001 (9)

### Geometric parameters (Å, º)

**Table:**

Cl1—C10	1.733 (2)	C6—C1	1.392 (3)
Cl2—C6	1.738 (2)	C8—C7	1.364 (3)
O1—C9	1.344 (2)	C8—H8	0.9300
O1—H1	0.8200	C13—H13	0.9300
N1—C13	1.268 (2)	C1—C2	1.362 (3)
N1—C3	1.424 (2)	C1—H1A	0.9300
C12—C7	1.390 (3)	C2—C3	1.394 (3)
C12—C11	1.392 (3)	C2—H2	0.9300
C12—C13	1.456 (3)	C4—C5	1.360 (3)
C9—C8	1.388 (3)	C4—C3	1.391 (3)
C9—C10	1.392 (3)	C4—H4	0.9300
C11—C10	1.368 (3)	C5—H5	0.9300
C11—H11	0.9300	C7—H7	0.9300
C6—C5	1.384 (3)		
			
C9—O1—H1	109.5	C2—C1—H1A	120.7
C13—N1—C3	120.11 (16)	C6—C1—H1A	120.7
C7—C12—C11	117.68 (18)	C11—C10—C9	120.60 (18)
C7—C12—C13	124.26 (18)	C11—C10—Cl1	120.45 (15)
C11—C12—C13	118.02 (17)	C9—C10—Cl1	118.90 (16)
O1—C9—C8	123.27 (17)	C1—C2—C3	122.28 (19)
O1—C9—C10	118.49 (17)	C1—C2—H2	118.9
C8—C9—C10	118.21 (18)	C3—C2—H2	118.9
C10—C11—C12	121.26 (18)	C5—C4—C3	121.8 (2)
C10—C11—H11	119.4	C5—C4—H4	119.1
C12—C11—H11	119.4	C3—C4—H4	119.1
C5—C6—C1	120.6 (2)	C4—C5—C6	119.4 (2)
C5—C6—Cl2	119.58 (16)	C4—C5—H5	120.3
C1—C6—Cl2	119.77 (16)	C6—C5—H5	120.3
C7—C8—C9	121.02 (19)	C8—C7—C12	121.22 (19)
C7—C8—H8	119.5	C8—C7—H7	119.4
C9—C8—H8	119.5	C12—C7—H7	119.4
N1—C13—C12	125.60 (19)	C4—C3—C2	117.33 (18)
N1—C13—H13	117.2	C4—C3—N1	125.25 (18)
C12—C13—H13	117.2	C2—C3—N1	117.42 (16)
C2—C1—C6	118.58 (19)		
			
C7—C12—C11—C10	−0.5 (3)	C8—C9—C10—Cl1	177.26 (15)
C13—C12—C11—C10	177.53 (18)	C6—C1—C2—C3	−0.3 (3)
O1—C9—C8—C7	178.76 (19)	C3—C4—C5—C6	0.4 (4)
C10—C9—C8—C7	0.5 (3)	C1—C6—C5—C4	−0.3 (4)
C3—N1—C13—C12	175.96 (17)	Cl2—C6—C5—C4	−179.65 (19)
C7—C12—C13—N1	−0.3 (3)	C9—C8—C7—C12	−0.8 (3)
C11—C12—C13—N1	−178.15 (18)	C11—C12—C7—C8	0.7 (3)
C5—C6—C1—C2	0.3 (3)	C13—C12—C7—C8	−177.13 (19)
Cl2—C6—C1—C2	179.62 (16)	C5—C4—C3—C2	−0.3 (3)
C12—C11—C10—C9	0.2 (3)	C5—C4—C3—N1	−179.3 (2)
C12—C11—C10—Cl1	−177.24 (16)	C1—C2—C3—C4	0.3 (3)
O1—C9—C10—C11	−178.58 (17)	C1—C2—C3—N1	179.32 (18)
C8—C9—C10—C11	−0.2 (3)	C13—N1—C3—C4	−7.7 (3)
O1—C9—C10—Cl1	−1.1 (3)	C13—N1—C3—C2	173.35 (18)

### Hydrogen-bond geometry (Å, º)

**Table:**

*D*—H···*A*	*D*—H	H···*A*	*D*···*A*	*D*—H···*A*
O1—H1···N1^i^	0.82	1.96	2.778 (2)	176

Symmetry code: (i) −*x*+1/2, *y*−1/2, −*z*+2.

## References

[bb20] Aydogan, F., Ocal, N., Turgut, Z. & Yolacan, C. (2001). *Bull. Korean Chem. Soc.* **22**, 476–480.

[bb1] Bernstein, J., Davis, R. E., Shimoni, L. & Chang, N.-L. (1995). *Angew. Chem. Int. Ed. Engl.* **34**, 1555–1573.

[bb2] Calligaris, M., Nardin, G. & Randaccio, L. (1972). *Coord. Chem. Rev.* **7**, 385–403.

[bb21] Cozzi, P. G. (2004). *Chem. Soc. Rev.* **33**, 410–421.

[bb22] Curini, A., Epifano, F., Maltese, F. & Marcotullio, M. C. (2002). *Tetrahedron Lett.* **43**, 3821–3823.

[bb23] Desai, S. B., Desai, P. B. & Desai, K. R. (2001). *Heterocycl. Commun.* **7**, 83–90.

[bb3] Farrugia, L. J. (1997). *J. Appl. Cryst.* **30**, 565.

[bb4] Farrugia, L. J. (1999). *J. Appl. Cryst.* **32**, 837–838.

[bb5] Flack, H. D. (1983). *Acta Cryst.* A**39**, 876–881.

[bb6] Gül, Z. S., Erşah˙in, F., Ağar, E. & Işık, Ş. (2007). *Acta Cryst.* E**63**, o2854.

[bb24] Karia, F. D. & Parsania, P. H. (1999). *Asian J. Chem.* **11**, 991–995.

[bb7] Lozier, R., Bogomolni, R. A. & Stoekenius, W. (1975). *Biophys. J.* **15**, 955–962. 10.1016/S0006-3495(75)85875-9 PMC1334761 1182271

[bb8] Şahin, Z. S., Ağar, A. A., Erşahin, F. & Işık, Ş. (2009). *Acta Cryst.* E**65**, o718. 10.1107/S1600536809007958 PMC2968940 21582454

[bb9] Şahin, O., Büyükgüngör, O., Albayrak, C. & Odabaşogˇlu, M. (2005). *Acta Cryst.* E**61**, o1288–o1290.

[bb10] Şahin, Z. S., Erşahin, F., Ağar, A. A. & Işık, Ş. (2009). *Acta Cryst.* E**65**, o547. 10.1107/S1600536809004784 PMC2968664 21582206

[bb11] Şahin, Z. S., Işık, Ş., Erşahin, F. & Ağar, E. (2009). *Acta Cryst.* E**65**, o811. 10.1107/S1600536809009684 PMC2968928 21582533

[bb12] Sheldrick, G. M. (2008). *Acta Cryst.* A**64**, 112–122. 10.1107/S0108767307043930 18156677

[bb13] Stoe & Cie (2002). *X-RED* and *X-AREA* Stoe & Cie, Darmstadt, Germany.

[bb14] Williams, D. R. (1972). *Chem. Rev.* **72**, 203–213. 10.1021/cr60277a001 4555559

